# Daily Temperature Effect on Seedling Growth Dynamic of Three Invasive Alien Species

**DOI:** 10.3389/fpls.2022.837449

**Published:** 2022-03-25

**Authors:** Maria Pepe, Loretta Gratani, Maria Fiore Crescente, Giacomo Puglielli, Laura Varone

**Affiliations:** ^1^Department of Environmental Biology, Sapienza University of Rome, Rome, Italy; ^2^Institute of Agricultural and Environmental Sciences, Estonian University of Life Sciences, Tartu, Estonia

**Keywords:** *Ailanthus altissima*, *Phytolacca americana*, *Robinia pseudoacacia*, relative growth rate, leaf production rate, leaf area production rate, air temperature

## Abstract

A greater relative growth rate (RGR) is positively correlated with a species’ ability to deploy a larger leaf area either due to a greater total number of leaves (LN) in the canopy or due to an average size of individual leaves (LA). This study aimed to analyze and compare, (1) the temporal (i.e., daily) RGR, leaf production rate (LPR), and leaf area production rate (LAPR) changes during the early growth stages of three among the most invasive species in the world, namely, *Ailanthus altissima*, *Phytolacca americana*, and *Robinia pseudoacacia*. (2) the interspecific differences in the relationship between RGR, LPR, LAPR, and mean daily air temperature. Our results show that growth dynamics as a function of temperature differ between invasive alien species (IAS). While these differences are partly explained by differences due to the growth form of the investigated species, the three IAS have a different behavior to adjust RGR, LPR, and LAPR with air temperature changes even within the same growth form, and in agreement with species habitat requirements in their native range. In conclusion, the results help disentangle the relative role of RGR, LPR, and LAPR in defining non-native species growth responses to mean daily air temperature also in relation to a species’ growth form.

## Introduction

Relative growth rate (RGR) is the result of a plant carbon economy and, therefore, one of the most widely used measures of plant performance (Lambers et al., 1989; Poorter and Lambers, 1991; Villar et al., 2005; Baraloto et al., 2006; Puglielli et al., 2017). At the cross-species level a greater RGR scale is positively correlated with a species’ ability to deploy a larger leaf area either due to a greater rate of production of leaves (LN) in the canopy [i.e., leaf production rate (LPR)] or due to a greater average size of individual leaves [LA; i.e., leaf area production rate (LAPR)] (Dijkstra and Lambers, 1986; Smeets and Garretsen, 1986; Poorter and Remkes, 1990; Atkin et al., 1996, 1998). Greater RGR, LPR, and LAPR are traits linked to faster acquisition and use of available resources that may confer a competitive advantage, especially in disturbed habitats (Grime and Hunt, 1975; Lambers and Poorter, 1992; Bellingham et al., 2004). For example, a rapid increase in the production of leaves (greater LPR) and/or a large increase in the rate of leaf area production (greater LAPR) might preempt the incoming solar radiation for overtopped vegetation (Grotkopp and Rejmánek, 2007). RGR, LPR, and LAPR show a high sensitivity to environmental factors, especially to air temperature (Weih, 2001; Zarzosa et al., 2021). Thus, the comparative analysis of RGR, LPR, and LAPR as a function of air temperature across species can inform on how species differentially adjust their growth performance to changing environmental conditions.

Despite the general interest in determining and comparing maximum (RGR_max_) across plant species, most of the studies have been generally carried out under controlled conditions (laboratory or greenhouse) (Villar et al., 2005), either using a single growth temperature or different static temperature treatments (e.g., Loveys et al., 2002). However, such a standardized procedure results in the lack of information on how RGR is dynamically adjusted with changing daily air temperature (Puglielli et al., 2017). Therefore, there is a general lack of knowledge in our understanding of both long- and short-term temperature responsiveness of RGR, LPR, and LAPR. This information might become particularly useful when trying to forecast species responses to some drivers of global climate change, e.g., increase in daily, seasonal, and annual mean temperatures.

Seedlings emergence and successful establishment are crucial phases of a species regeneration niche (Grubb, 1977), and they directly affect plant’s chances to reach the reproductive phase (Moravcová et al., 2006; Vandelook and Van Assche, 2008; Morimoto et al., 2010; Cochrane et al., 2015). Thus, the size and persistence of plant populations ultimately depend on seedling emergence and survival (Kitajima and Fenner, 2000; Grime, 2001) dictated by species’ habitat preferences and rhythm of life cycle events (Nikolaeva, 1999; Vandelook and Van Assche, 2008). There is a large consensus in recognizing traits, including high RGR and total leaf area, as key traits related to seedling’s ability to successfully establish and occupy free niche space (van Kleunen et al., 2010; Dawson et al., 2011; Richardson and Pyšek, 2012; Moodley et al., 2013). The self-sustaining populations are important traits of plants for their process of becoming invasive alien species (IAS). Much interest has therefore been shown in analyzing interspecific and intraspecific differences in RGR among IAS (Dawson et al., 2011; Li et al., 2016).

The IAS are generally introduced by humans in a conscious or not manner, in areas outside their natural distribution range. However, not all species behave similarly once introduced into a new territory. Some species do not survive during transport, whereas other species cannot adapt to the new abiotic and biotic conditions adapt to new conditions. Some remain in confined environments (e.g., cultivated species) while others survive but are not able to create persistent nuclei. On the other hand, other introduced species are able to adapt to the new environment and reproduce (i.e., non-native plants). Among the non-native plants, some have the ability to colonize the territory in a short time and become prevalent within the new area causing harm to the environment and other species. These species are referred to as IAS (AA.VV, 2007; Winter et al., 2009). IAS are now considered one of the most severe threats to biodiversity, because (i) they reduce native species richness and abundance (Olden and Poff, 2003; Sax and Gaines, 2003; Winter et al., 2009); (ii) they alter the genetic structure of native populations *via* hybridization (Vilà et al., 2000); and (iii) they disrupt mutualistic networks, e.g., pollination (Traveset and Richardson, 2006; Schweiger et al., 2010; Pyšek et al., 2012).

Many non-native plants arrive from regions with a warmer climate than the climate of the new areas where they (Walther et al., 2009). Therefore, studying interspecific differences in the growth behavior of non-native species as a function of varying air temperature might inform on how these species respond to shifts in temperature regimes due to the introduction process, and ultimately due to climate change.

However, as outlined above, the early life cycle stages of a plant are also highly sensitive to temperature changes (Bond and van Wilgen, 1996; Cochrane et al., 2015). Thus, understanding IAS seedlings’ growth dynamics, i.e., changes in RGR, LPR, and LAPR, in response to temperature changes, can greatly contribute to improve our understanding of IAS growth dynamics under new temperature regimes, as those imposed by global climate change. Despite that, we still lack a clear understanding of how IAS growth dynamics are influenced by changing temperature.

In this context, this study aimed to analyze and compare (1) the temporal (i.e., daily) RGR, LPR, and LAPR changes during the early growth stages of three among the most invasive species in the world, namely, *Ailanthus altissima* (Mill.) Swingle (AA), *Phytolacca americana* L. (PA), and *Robinia pseudoacacia* L. (RP) and (2) the interspecific differences in the relationship between RGR, LPR, LAPR, and mean daily air temperature. Moreover, given that these species have different temperature preferences in their sites of origin, we discussed how temporal variations in seedling RGR, LPR, and LAPR in response to temperature can reflect, or not, the species habitat requirements in their native range.

## Materials and Methods

### Plant Species

The presence of IAS is particularly significant in Europe, where their number has increased fourfold in the last century (Hulme et al., 2009). Among others, AA, PA, and RP are the most invasive plant species in Europe, and they are particularly spread in urban areas. These species were introduced essentially for ornamental and economic purposes between XVII and XIV centuries in Europe (Kowarik and Säumel, 2007; Balogh and Juhász, 2008; Cierjacks et al., 2013), and their spreadability has substantially been increased due to their capacity to establish in ever-increasing areas disturbed by human intervention.

The AA is a tree species native to subtropical/warm temperate climates but it is able to invade climates ranging from cool temperate to tropical. The preferred mean annual temperature is 7–18°C, and it can also tolerate heavy frosts (CABI, 2021). In its invaded range, the species is common in urban or disturbed areas, but in the Mediterranean region, it also occurs in semi-natural habitats with increasing negative impacts on native outcompeted species (Gómez-Aparicio and Canham, 2008; Constán-Nava et al., 2010; Petruzzellis et al., 2019).

The PA is an herbaceous perennial species occurring rarely on sites where the temperature is negative for long periods in winter. In particular, the propagation is favorable if the average temperature is around 20°C in July (Balogh and Juhász, 2008). This species mostly germinates in disturbed soils and on sunny and shady sites alike. In Italy, it was found on field sides, along canals, on the seaside, and in black locust plantations (Balogh and Juhász, 2008).

The RP is a tree species and, in its native range, grows best at sites characterized by a humid climate with mean temperatures in January from 4 to 7°C and in August from 18 to 27°C (Huntley, 1990; Cierjacks et al., 2013). It is now well-established in the southern parts of the British Isles and continental Europe (Clement and Foster, 1994; Kowarik and Rabitsch, 2010; Cierjacks et al., 2013).

### Study Site and Plant Material

Measurements were carried out in the period of February–July 2019 at the Botanical Garden of the Sapienza University of Rome (41°53′ N, 12°28′ E; 53 m a.s.l.). Freshly matured seeds of AA, PA, and RP were collected from plants naturally growing in different public sites, comparable with conditions, in the city of Rome (Italy) at the beginning of October 2018, after having obtained permission from the local authority. Collection of seeds and study were conducted according to the local and national regulations.

The climate of Rome is of Mediterranean type, and most of the total annual rainfall (850.8 mm) occurs in autumn and winter (Data from Arsenal Meteorological Station, Lanciani Street 2009–2019). The mean minimum air temperature of the coldest month (January) was 4.6°C, the mean maximum air temperature of the hottest month (August) was 32.4°C, and the annual mean air temperature was 17.0°C. The dry period was from June to August (106.4 mm of total rainfall during that period).

Seeds were stored in paper bags under room conditions until the beginning of the experiment. Sowing was carried out in January 2019, and seedlings, which emerged in February, were cultivated in an open space and were stored in black polyethylene plastic pots (with 14 cm diameter, 16 cm height, and 2.5 L). The pots were filled with a basic cultivation substrate (COMPO Naturasol Universal, Italy) with the following composition: neutral sphagnum peat, composted green soil improver, pumice, pH (H_2_O) 7.0, electrical conductivity (dS m^–1^) 0.60, dry bulk density (kg m^–3^) 220, and total porosity (% v/v) 88%. Plants were regularly watered during the entire duration of the measurements.

### Growth Analysis

Twenty-three seedlings per species were monitored every 5 days in the period of February–July 2019. At each sampling day, the following parameters were monitored: seedling height (H, cm), defined as the major distance from the soil level to the highest point of the plant; the LN (*n*); and the LA (cm^2^; seven leaves per species) determined using the Image Analysis System (Delta-T Devices, Cambridge, United Kingdom). Measurements were carried out until no significant differences (*p* ≤ 0.05) in H among species were observed.

We then identified the best non-linear function describing the relationship between log_10_-transformed H, LN, and LA as a function of time for all data pooled using the Curve Finder function of CurveExpert 1.4 (Hyams Development, Chattanooga, Tennessee, United States). This function employs many regression models, and each fit is ranked according to its standard error and correlation coefficient. High-order (>3) polynomials were excluded to avoid overfitting. The three-parameter logistic function was found to be the best fit for all the parameters, as described by Puglielli et al. (2017). After having determined the functional form of the model, parameters were estimated using 50-fold cross-validation on random subsets of the original dataset stratified per species. At each run, the model was calibrated on 70% of the input data and its predictive accuracy was evaluated on the remaining 30% by simple linear regression (i.e., correlation coefficient between predicted vs. observed values). At each run, model parameters were separately estimated for each species-trait combination using the nlsList function (nlme R package; Pinheiro et al., 2020). Mean and standard deviation for model parameters after cross-validation and their correlation coefficient predicted vs. observed values are summarized in Supplementary Table 1.

The RGR in plant height (cm cm^–1^ day^–1^), LPR (n n^–1^ day^–1^), and LAPR (cm^2^ cm^–2^ day^–1^) were calculated as the derivative with respect to time of the cross-validated models, as proposed by Paine et al. (2012).

### Temperature Dependence of Growth Parameters

Once the daily values of RGR, LPR, and LAPR were obtained, we tested their relationship with temperature as follows:

(1) We calculated the mean daily air temperature by averaging hourly temperature data on a 24 h basis. Temperature data were recorded using the HOBO data logger (H08-003-02; Onset HOBO Data Loggers, Cape Cod, MA, United States).

(2) We evaluated the relationship between daily values of RGR, LPR, and LAPR and mean daily air temperature using the Curve Finder function of CurveExpert 1.4. The best model describing the relationship for all the parameters was a Gaussian model in the form:


$$\mathrm{Growth}\text{parameter}=ae^{-{\left(b-T\right)}^{2}/\left(2c^{2}\right)}$$

where *T* represents mean daily air temperature. Model parameters were estimated per trait-temperature-species combination using 10-fold cross-validation as already described. We chose a lower number of iterations to account for the lower number of data points for this analysis.

(3) To analyze the temperature-dependent behavior of the considered traits among species, we selected three target temperatures (i.e., 13, 18, and 23°C), corresponding to temperatures at which the maximum, intermediate, and minimum values of the considered traits were measured. We then applied one-way ANOVA to evaluate trait differences among species at each target temperature.

Cross-validation curves (Gaussian model) for the relationships among RGR, LPR, and LAPR and mean daily air temperature for each species are shown in Supplementary Figure 1. In this figure, each intersection between curves and vertical dashed line represents a data point used to draw Figure 2 All the analyses were conducted using R version 4.1.1 (R Core Team, 2021).^1^

## Results

### Growth Analysis

**FIGURE 1:**
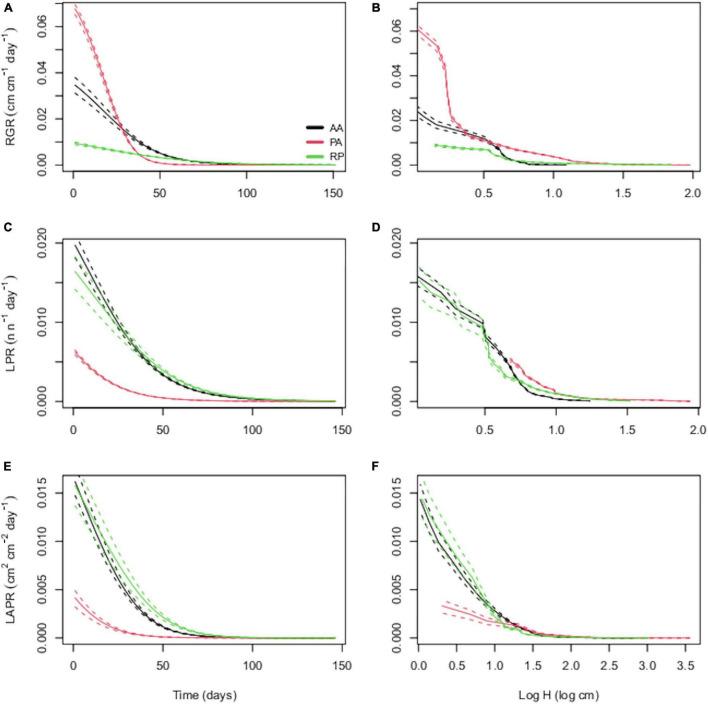
Temporal trends of relative growth rate [relative growth rate (RGR); cm cm^–1^ day^–1^], leaf production rate (LPR; n n^–1^ day^–1^), and leaf area production rate (LAPR; cm^2^ cm^–2^ day^–1^) for *Ailanthus altissima* (AA), *Phytolacca americana* (PA), and *Robinia pseudoacacia* (RP). In panels **(A,C,E)**, the derivation of considered parameters as a function of time was shown, while in panels **(B,D,F)**, the considered parameters were related to seedling height (H).

The output of the cross-validation for the relationship among H, LN and LA and time showed correlation coefficients of predicted-observed values in the range 0.71–0.80 (Supplementary Table 1). Despite the similar temporal trends of RGR, LPR, and LAPR across species (Figures 1A,C,E), the interspecific differences at any single time step largely reflected the differences between herbaceous PA and woody AA and RP. In particular, PA showed the greatest RGR followed by AA and RP (Figure 1A), and this difference is maintained for approximately 40 days. Conversely, in the same time range, PA showed the lowest LPR and LAPR. AA and RP showed higher LPR and LAPR values than PA but with a considerable overlap for these traits among them (Figures 1A,C,E). The same differences were maintained when species were compared at common H (Figures 1B,D,F).

### Temperature Dependence of Growth Parameters

**FIGURE 2:**
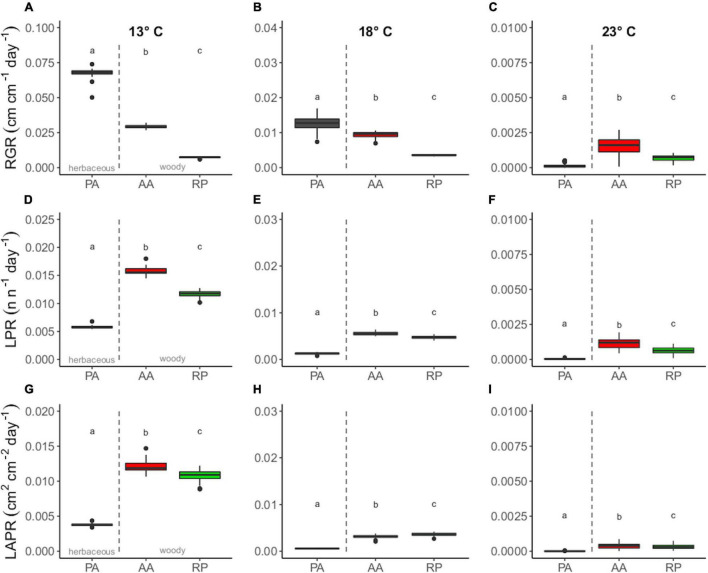
Boxplot of panels **(A–C)** relative growth rate (RGR) (cm cm^–1^ day^–1^), **(D–F)** LPR (n n^–1^ day^–1^), and **(G–I)** LAPR (cm^2^ cm^–2^ day^–1^) for *Ailanthus altissima* (AA), *Phytolacca americana* (PA), and *Robinia pseudoacacia* (RP) at three target temperatures. Differences among species were evaluated at 13–18 and 23°C. Different letters indicate significant differences (one-way ANOVA, *p* ≤ 0.05).

Concerning the response of the species at the three target temperatures, PA showed the greatest mean RGR at 13 and 18°C (0.0669 ± 0.0014 and 0.0133 ± 0.0025 cm cm^–1^ day^–1^), followed by AA (0.0293 ± 0.0012 and 0.0095 ± 0.0009 cm cm^–1^ day^–1^) and RP (0.0075 ± 0.0003 and 0.0036 ± 0.0002 cm cm^–1^ day^–1^) (Figures 2A,B). Moreover, differences among the species tended to vanish at 23°C, mostly due to arrested growth (Figure 2C). However, PA showed the lowest RGR at this temperature compared to AA and RP.

AA and RP showed the greatest mean values of LPR and LAPR at any given target temperature (Figures 2D,G). Cross-species differences for the considered parameters among species were significant at any given temperature were found among species at the three target temperatures (Figures 2A–I).

## Discussion

Our results show a common temporal dynamic of the growth parameters among the considered species, which reached the highest RGR, LPR, and LAPR approximately 40 days from germination. Nevertheless, at the early time steps, PA displayed the greatest RGR_max_ associated with the lowest LPR and LAPR, whereas AA and RP had an opposite trend. The behavior of PA reveals a typical herbaceous growth strategy characterized by a greater RGR_max_ for plant height compared to the woody AA and RP. The hollow stems of herbaceous species are in fact often short-lived with reduced requirement for mechanical support, compared to woody species (Sun and Frelich, 2011), possibly resulting in a greater RGR_max_ of PA compared to AA and RP.

Conversely, LPR and LAPR were the greatest for AA and RP. These traits are strictly related to plant capacity for light capture and use (Grotkopp and Rejmánek, 2007; Niinemets, 2010). The capacity of AA and RP to invest more resources for increasing LPR and LAPR rather than RGR_max_, might reflect a seedling growth strategy aimed at optimizing competition for light capture. AA and RP generally exhibit a preference for establishing in heavily disturbed areas where the plant canopy is not dense, revealing a better performance under high light conditions (Godefroid and Ricotta, 2018; Granata et al., 2020). In support of this, AA is generally defined as a highly shade-intolerant species whose successful establishment depends on disturbed areas with full light (Kowarik, 1995; Fotiadis et al., 2011). The highest seedlings LPR and LAPR might in fact allow AA and RP to occupy relatively large spaces through a rapid increase in canopy width and denseness during their early growth stages, and this strategy is compatible with an efficient exclusion of other species from beneath their expanding canopy.

PA showed a strategy based on a greater investment in RGR aimed at maximizing vertical growth. This could confer a competitive advantage to effectively compete for light-harvesting with coexisting species during early growth stages (Pearcy et al., 2004; Valladares and Niinemets, 2008; Niinemets, 2010). Moreover, a high RGR is frequently positively associated with high annual biomass production (van Kleunen et al., 2010). Thus, the RGR maximization behavior in PA could mirror a higher resource allocation to the physiological components of RGR, such as the net assimilation rate (Lambers et al., 2008). In contrast, our results support the idea that AA and RP probably enhance their success during the early growth stages by maximizing plant leafiness, a crucial feature to increase space occupancy and possibly fitness in highly for woody plants.

Concerning the interspecific differences in RGR at the target temperatures, the response of PA could reflect a narrower temporal window to maximize its growth in height compared to AA and RP. On the other hand, AA and RP seem to have a larger temporal window than PA for growing in height. However, some differences between AA and RP exist as well. In particular, AA maintained a greater LPR at any temperature than RP, while RP showed a greater LAPR for a longer time than AA. This difference between AA and RP may reflect interspecific differences in seedling architecture, habitat preferences in the native range of these species, or both. The interaction between plant architecture and species habitat preferences can have an important effect on plant traits, e.g., on biomass allocation to leaves, stems, and roots (Puglielli et al., 2021).

Finally, we argue that a greater LAPR at all temperatures for RP compared to AA might permit the more shade-tolerant RP to maximize LAPR as soon as the temperature is favorable enough for this process, possibly before the canopy of the dominant layer closes. AA, which is instead shade-intolerant, might seek a shade avoidance strategy growing faster than RP in terms of height, possibly linked to the production of a multilayered crown, typical of shade avoidant species (Niinemets, 2010). This statement is supported by the greater LPR of AA, not mirrored by greater LAPR, at almost all temperatures, compared to RP.

Given our results, it is tempting to speculate that the patterns we found could reflect species’ adaptations to the climatic conditions in their native habitats. In particular, in the case of woody species, AA comes from subtropical or warm temperate climates with a long and warm growing season (Kowarik and Säumel, 2007). Accordingly, at each of the considered temperature, AA showed a greater RGR than RP, which is the only species whose range includes the cool temperate moist forest and warm temperate montane moist forest (Sawyer and Lindsey, 1963; Huntley, 1990). Conversely, PA rarely grows in sites characterized by cold temperatures in winter (Balogh and Juhász, 2008), and this was already proposed as a general characteristic of the species belonging to Phytolaccaceae family (USDA, 2022; Balogh and Juhász, 2008). This suggests that PA growth might strongly depend on the breadth of the temperature window in which growth can be maximized, consistent with the faster vertical growth we found compared to the other species. In line with this, Pepe et al. (2020) also found that PA germination capability is enhanced at higher temperatures compared to AA and RP.

## Conclusion

We have shown that RGR, LPR, and LAPR and mean daily air temperature directly informs about the habitat requirements of the species. In particular, the results help disentangle the role of RGR, LPR, and LAPR for non-native species in coping with air temperature changes especially when compared with similar studies conducted of native species (Puglielli et al., 2017). Our analysis can, therefore, widen our understanding of the interspecific differences in IAS growth strategies during the early phases of their establishment on the basis of species’ growth form, the considered traits, and possibly their trade-offs.

## Data Availability Statement

The raw data supporting the conclusions of this article will be made available by the authors, without undue reservation.

## Author Contributions

MP conceived the idea, contributed to design the experiment, coordinated and performed the field data collection, performed the data analysis, and led the writing of the manuscript. LG contributed to the writing of the manuscript with constructive suggestions. MC coordinated the collection of field data and contributed to the writing of the manuscript. GP contributed to conceptualization, planned, and led the data analysis. LV conceived the idea, designed the experiment, and led the writing of the manuscript. All authors contributed critically to the final manuscript and gave their approval for publication.

## Conflict of Interest

The authors declare that the research was conducted in the absence of any commercial or financial relationships that could be construed as a potential conflict of interest.

## Publisher’s Note

All claims expressed in this article are solely those of the authors and do not necessarily represent those of their affiliated organizations, or those of the publisher, the editors and the reviewers. Any product that may be evaluated in this article, or claim that may be made by its manufacturer, is not guaranteed or endorsed by the publisher.

## Footnotes

1
https://www.r-project.org/
