# Supplementary figures and images for: Daily Temperature Effect on Seedling Growth Dynamic of Three Invasive Alien Species
Source: Front Plant Sci. 2022 Mar 25;13:837449. doi: 10.3389/fpls.2022.837449 (PMC8990299; doi:10.3389/fpls.2022.837449)

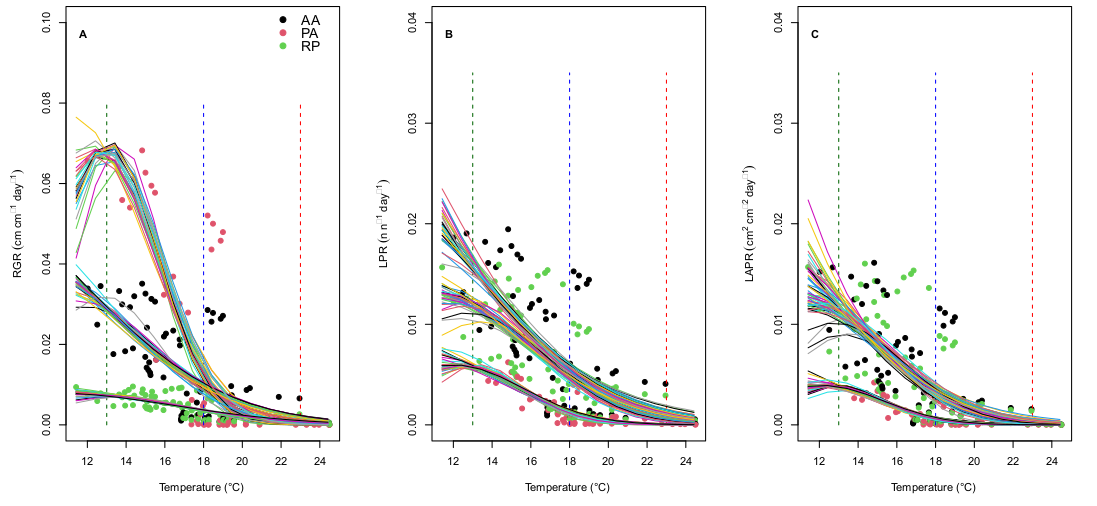

Supplement: Supplementary file 1 [file Image_1.tiff]
