# Supplementary material for: Daily Temperature Effect on Seedling Growth Dynamic of Three Invasive Alien Species
Source: Front Plant Sci. 2022 Mar 25;13:837449. doi: 10.3389/fpls.2022.837449 (PMC8990299; doi:10.3389/fpls.2022.837449)
Supplement: Supplementary file 2 [file Table_1.DOCX]

Supplementary Material

# Supplementary Tables

|  | **Relative Growth Rate (RGR)** | | | | |  | **Leaf production rate (LPR)** | | | | |  | **Leaf area production rate (LAPR)** | | | | |
| --- | --- | --- | --- | --- | --- | --- | --- | --- | --- | --- | --- | --- | --- | --- | --- | --- | --- |
|  | a_cv_ | b_cv_ | c_cv_ | R_cv_ | n_cv_ |  | a_cv_ | b_cv_ | c_cv_ | R_cv_ | n_cv_ |  | a_cv_ | b_cv_ | c_cv_ | R_cv_ | n_cv_ |
| AA | 0.073 _(0.096)_ | 5.949 _(6.187)_ | 6.278 _(1.918)_ | 0.802 _(0.077)_ | 57.0 _(0.04)_ |  | 0.050 _(0.083)_ | 4.014 _(7.241)_ | 7.158 _(2.077)_ | 0.741 _(0.054)_ | 56.3 _(0.05)_ |  | 0.068 _(0.219)_ | 5.506 _(6.845)_ | 5.820 _(1.875)_ | 0.711 _(0.057)_ | 56.3 _(0.05)_ |
| PA | 0.068 _(0.001)_ | 12.748 _(0.517)_ | 2.268 _(1.901)_ | 0.804 _(0.078)_ | 42.7 _(0.06)_ |  | 0.06 _(0.001)_ | 11.756 _(1.050)_ | 3.491 _(0.570)_ | 0.749 _(0.059)_ | 43.2 _(0.06)_ |  | 0.004 _(0.001)_ | 11.953 _(1.102)_ | 3.073 _(0.525)_ | 0.714 _(0.065)_ | 42.4 _(0.06)_ |
| RP | 0.008 _(0.001)_ | 11.134 _(1.146)_ | 5.398 _(0.650)_ | 0.803 _(0.077)_ | 63.3 _(0.05)_ |  | 0.013 _(0.001)_ | 11.296 _(1.316)_ | 4.783 _(0.744)_ | 0.749 _(0.059)_ | 62.4 _(0.04)_ |  | 0.012 _(0.002)_ | 11.189 _(1.392)_ | 4.355 _(0.694)_ | 0.714 _(0.065)_ | 63.3_(0.04)_ |

**Supplementary Table 1.** Model parameters after cross-validation for the relationship between Relative Growth Rate (RGR), leaf production rate (LPR), leaf area production rate (LAPR) and temperature for species. Parameters with subscript are the one obtained after cross validation and mean and standard deviation (in parenthesis) per each coefficient is shown. R_cv_ = correlation coefficient between Predicted values after cross-validation vs. Observed values. n_cv_ = mean number of observations within each species and its coefficient of variation (in parenthesis) across 10 runs. n = 10. AA = *Ailanthus altissima*; PA = *Phytolacca americana*; RP = *Robinia pseudoacacia*. Curves from which the parameters were obtained are shown in Supplementary Figure 1.

# Supplementary Figures

**Supplementary Figure 1. Cross-validation curves across species.** All the curves (Gaussian model) obtained from cross validation procedure describing the relationship between (A) Relative Growth Rate (RGR, cm cm^-1^ day^-1^), (B) leaf production rate (LPR, n n^-1^ day^-1^) and (C) leaf area production rate (LAPR, cm^2^ cm^-2^ day^-1^) and temperature (°C). Vertical dashed lines represent the temperatures (13–18–23 °C) at which differences between species were evaluated. Each intersection between curves and vertical dashed line represent a data point used to draw Fig. 2 in the main text. Note that x-axis range is reduced compared to previous figures to better show the temperature at which differences among species were evaluated.
